# Integrated Transcriptomics Establish Macrophage Polarization Signatures and have Potential Applications for Clinical Health and Disease

**DOI:** 10.1038/srep13351

**Published:** 2015-08-25

**Authors:** Matheus Becker, Marco A. De Bastiani, Mariana M. Parisi, Fátima T. C. R. Guma, Melissa M. Markoski, Mauro A. A. Castro, Mark H. Kaplan, Florencia M. Barbé-Tuana, Fábio Klamt

**Affiliations:** 1 Laboratory of Cellular Biochemistry, Department of Biochemistry, ICBS/UFRGS, 90035-003 Porto Alegre (RS), Brazil; 2 National Institutes of Science & Technology—Translational Medicine (INCT-TM), 90035-903 Porto Alegre (RS), Brazil; 3 Laboratory of Molecular Biology and Bioinformatics, Department of Biochemistry, ICBS/UFRGS, 90035-003 Porto Alegre (RS), Brazil; 4 Laboratory of Biochemistry and Cellular Biology of Lipids, Department of Biochemistry, ICBS/UFRGS, 90035-003 Porto Alegre (RS), Brazil; 5 Laboratory of Cellular and Molecular Cardiology, IC/FUC, Porto Alegre, RS 90620-000, Brazil; 6 Laboratory of Bioinformatics, Professional and Technological Education Sector, Polytechnic Center, UFPR, 81531-970 Curitiba (PR), Brazil; 7 Department of Pediatrics, Herman B Wells Center for Pediatric Research, Indianapolis (IN), 46202, USA; Department of Microbiology and Immunology, Indiana University School of Medicine, Indianapolis (IN), 46202, USA; 8 Biomedical Research Institute, PUCRS, 90619-900, Porto Alegre (RS), Brazil

## Abstract

Growing evidence defines macrophages (Mφ) as plastic cells with wide-ranging states of activation and expression of different markers that are time and location dependent. Distinct from the simple M1/M2 dichotomy initially proposed, extensive diversity of macrophage phenotypes have been extensively demonstrated as characteristic features of monocyte-macrophage differentiation, highlighting the difficulty of defining complex profiles by a limited number of genes. Since the description of macrophage activation is currently contentious and confusing, the generation of a simple and reliable framework to categorize major Mφ phenotypes in the context of complex clinical conditions would be extremely relevant to unravel different roles played by these cells in pathophysiological scenarios. In the current study, we integrated transcriptome data using bioinformatics tools to generate two macrophage molecular signatures. We validated our signatures in *in vitro* experiments and in clinical samples. More importantly, we were able to attribute prognostic and predictive values to components of our signatures. Our study provides a framework to guide the interrogation of macrophage phenotypes in the context of health and disease. The approach described here could be used to propose new biomarkers for diagnosis in diverse clinical settings including dengue infections, asthma and sepsis resolution.

Macrophages (Mφ) are pivotal cells of the immune system that participate in pleiotropic actions^1^. Microenvironmental signals promote the development of Mφ subsets that secrete specific cytokines and perform distinct functions in regulating and resolving immunity, perpetuation of inflammation^2^,^3^,^4^,^5^, or as lately suggested regulating blood supply and metabolism^6^,^7^. Sub-populations of Mφ exist within a continuum of diverse interchangeable phenotypic spectrums designated in the literature for simplicity as M1 (classically activated), or M2a, M2b and M2c (collectively termed alternatively activated). They have overlapping functions that can be modulated by inducers including hematopoietic growth factors and cytokines (*e.g*.: Macrophage Colony Stimulating Factor (M-CSF) or Granulocyte Macrophage Colony Stimulating Factor (GM-CSF)) or small metabolites such as glucose, lipids or sodium chloride^2^,^8^,^9^,^10^,^11^,^12^,^13^. To add more complexity to this issue, Mφ exhibit plasticity across various diseases and can switch their phenotype depending on the *in vivo* environment and stage of the disease. As an example, more than 9 different gene signatures based on distinct spectrum of transcriptional programs have been described recently for human macrophages^14^. Moreover, the inconsistency found in the diversity of terminology, activation inducers and markers used to describe Mφ subsets, enhance the complexity when comparisons of different studies are required for consensus^15^.

Since different Mφ subsets are profoundly involved in the development and outcome of many of the so called “Western diseases” (*e.g.*: autoimmune diseases, atherosclerosis, cancer, microorganisms infections and asthma) and are key cells in controlling normal physiological processes^2^,^5^,^16^,^17^,^18^,^19^,^20^, here we question whether a restricted set of marker molecules could be helpful to define a particular functional phenotype encountered in the context of diseases. With this in mind, the presence of a minimum set of specific markers that describe a particular phenotype dependent on the inducers used to generate the Mφ, could be seen as a valuable tool to classify and study defined Mφ subsets found in a specific context, in order to develop precise targeted Mφ immunotherapies.

In the present study we explored the use of bioinformatics’ tools to analyze published transcriptome data in predefined *in vitro* conditions for Mφ activation. A robust phenotype signature, herein named M(IFNγ + LPS, TNFα) and M(IL-4, IL-13), was obtained from the analysis of responsive genes in three pre-selected datasets where human monocyte-derived macrophages (MDM) were challenged under classical activators (IFNγ + LPS, TNFα) or alternative inducers (IL-4 or IL-13). The expression of some selected markers was confirmed by real time quantitative polymerase chain reaction (RT-qPCR) in *in vitro* MDM derived from healthy human peripheral blood mononuclear cells (PBMC) and in commonly used differentiated human cell lines (THP-1 and U-937). Finally, we validated our list using independent original microarray datasets of clinical cohorts in the context of different diseases. In this regard, we were able to attribute a minimum set of molecular biomarkers that corresponded to defined Mφ phenotypes among milieus of specific diseases. Our signatures effectively identified classically M(IFNγ + LPS,TNFα) and alternatively M(IL-4, IL-13) activated Mφ in clinical controlled sets. More importantly, we demonstrated prognostic and predictive values of selected biomarkers associated with diseases in diverse clinical settings such as dengue infections, asthma and sepsis resolution.

## Results

### Generation of M(IFNγ + LPS,TNFα) and M(IL-4, IL-13) Gene Signatures

**Figure 1:**
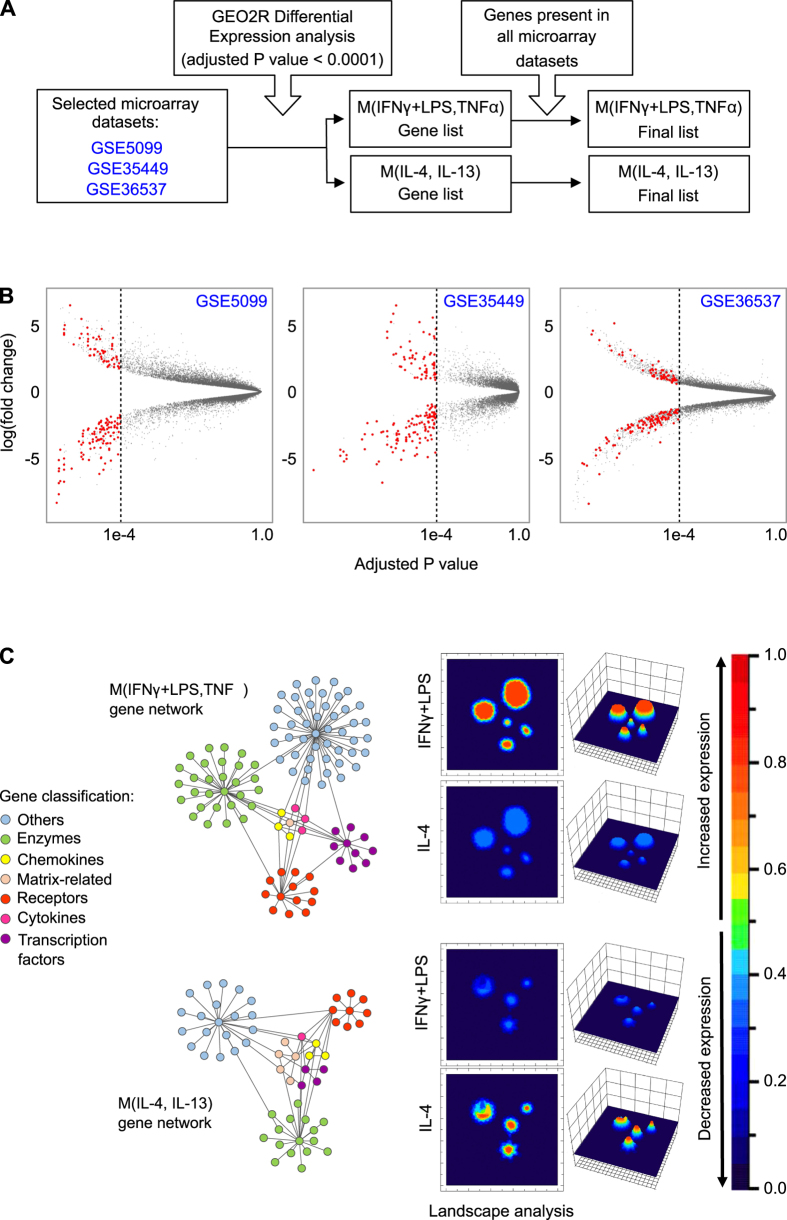
Macrophage phenotypes signatures construction and gene network representation. (**A**) Protocol design for M(IFNγ + LPS, TNFα) and M(IL-4, IL-13) gene signatures. (**B**) Volcano plots representation of differential expression analyses. Red dots are genes present in all three datasets with adjusted P value ≤0.0001. (**C**) M(IFNγ + LPS, TNFα) and M(IL-4, IL-13) gene networks (left) and their illustrative topological representation (landscape analysis) showing changes in relative gene expression after IFNγ + LPS or IL-4 stimuli (right) (see Supplementary Table S2 & S3 for the complete list of retrieved genes).

Heterogeneous sources of cells, experimental inducers and markers are used to describe phenotypes and responses of polarized Mφ creating an enormous amount of conflicting data^15^. To systematically evaluate data from defined experimental conditions, as classically and alternatively activated Mφ, we specifically chose *in vitro* datasets reporting explicit description of experimental standard conditions. In this regard, we integrated gene expression profiling from three independent human datasets (*GSE5099*^21^; *GSE35449*^22^; *GSE36537*^23^) that used peripheral blood mononuclear cells as the source for differentiation into MDM with M-CSF and polarized *in vitro* with IFNγ + LPS or TNFα, IL-4 or IL-13 (Supplementary Table S1). The protocol design is illustrated in Fig. 1A.

Using *GEO2R* tool analysis in each selected dataset we obtained two differentially expressed gene signatures that we termed the M(IFNγ + LPS, TNFα) and M(IL-4, IL-13) phenotypes (Fig. 1B), following the guidelines recently suggested by Murray *et al.*^15^. Afterward, with an inclusion criteria of *P* value ≤ 0.0001 (Fig. 1B, vertical line is the cut-off) and presence in all three datasets (red dots in Fig. 1B), our selection resulted in 106 M(IFNγ + LPS, TNFα) and 58 M(IL-4 or IL-13) differentially expressed genes (Supplementary Table S2 and S3). Finally, signatures of both phenotypes were depicted as graphic models with nodes representing gene products, clustered according to seven previously reported functional subdivisions^15^. (Fig. 1C). These gene signatures are comprised of established enzymes (*ALOX15*), cytokines (*TNFα* and *TGFβ*), chemokines (*CCL13*, *CCL17*, *CXCL9* and *CXCL10*), receptors and transcription factors (*STAT1*), and less explored genes such as LAG-3 (lymphocyte-activation gene 3), LAMP3 (lysosomal-associated membrane protein 3), *FZD2* (frizzled class receptor 2) and *CLIC2* (chloride intracellular channel 2) among others. In order to illustrate differential gene expression responses to inducers, the obtained M(IFNγ + LPS, TNFα) and M(IL-4, IL-13) gene networks were subsequently plotted as representative topological responses using landscape analysis with ViaComplex^®^ software^24^. *X* and *y*-axis represent gene signature networks and the *z*-axis indicates an illustrative expression response to IFNγ + LPS or IL-4 inducers (Fig. 1C).

### *In Vitro* Validation of M(IFNγ + LPS, TNFα) and M(IL-4, IL-13) Gene Signatures

**Figure 2:**
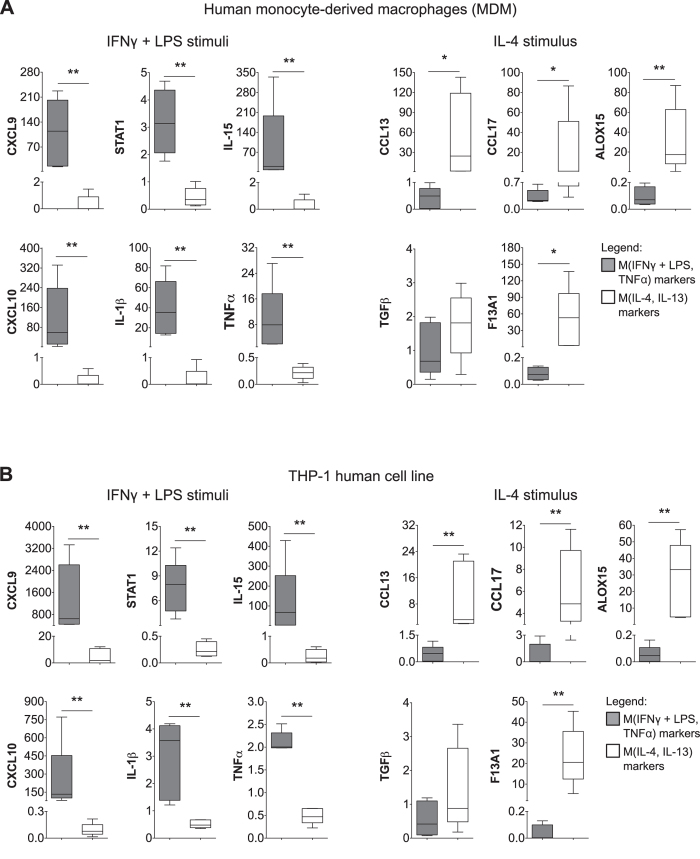
*In vitro* validation of selected genes from M(IFNγ + LPS, TNFα) and M(IL-4, IL-13) signatures. (**A**) RT-qPCR from human MDM activated with 50 ng/mL of M-CSF for 7 days and stimulated with IFNγ (20 ng/mL) + LPS (100 ng/mL) or IL-4 (20 ng/mL) for additional 18 h. (**B**) RT-qPCR from THP-1 (human acute monocyte leukemia cell line) differentiated with 20 ng/mL PMA for 3 days and stimulated with IFNγ (20 ng/mL) + LPS (100 ng/mL) or IL-4 (20 ng/mL) for additional 24 h. Data represent median and IQR (interquartile range) of five independent experiments normalized to TATA binding box protein (TBP). Data was considered statistically significant for *(P ≤ 0.05) and ** (P ≤ 0.01) (Mann-Whitney U test).

To experimentally validate our *in silico* Mφ signatures, we used standard M-CSF conditions to differentiate human Mφ from peripheral CD14^ +^ blood monocytes obtained from five healthy donors. These cells were activated/polarized into the M(IFNγ + LPS) or M(IL-4) phenotypes. Figure 2A (left) shows RT-qPCR results from MDM stimulated with IFNγ + LPS. In agreement with the literature, we observed increased expression of selected targets of M(IFNγ + LPS, TNFα), such as *CXCL9*, *CXCL10*, *IL-1β*, *IL-15, STAT1* and *TNFα*. In the same way, RT-qPCR analysis of IL-4-induced MDM revealed increased expression of the selected targets of M(IL-4, IL-13), such as *ALOX15*, *CXCL13*, *CXCL17* and *F13A1*, with no significant upregulation of *TGFβ* (Fig. 2A). Similar results were obtained from the cultured THP-1 cell line (Fig. 2B) and U-937 cells (manuscript in preparation).

### Application of M(IFNγ + LPS, TNFα) and M(IL-4, IL-13) Signatures to Discriminate Clinical Settings and Controlled Conditions

**Figure 3:**
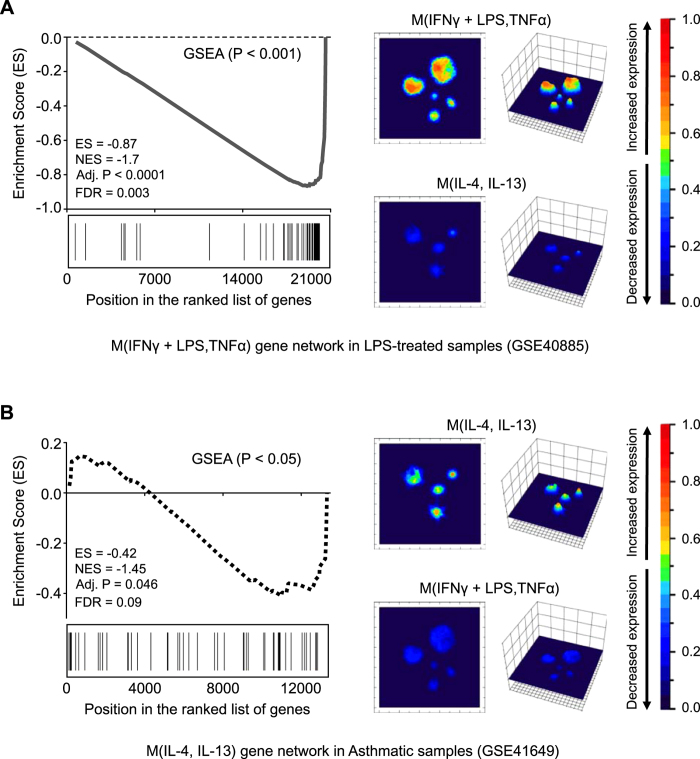
Validation of M(IFNγ + LPS, TNFα) and M(IL-4, IL-13) signatures under controlled clinical settings. (**A**) M(IFNγ + LPS, TNFα) signature response of alveolar macrophages after LPS instillation in the lung based on Gene Set Enrichment Analysis (GSEA) (left) and topological representation (landscape analysis) (right). (**B**) M(IL-4, IL-13) signature response of bronchial biopsy from asthmatic patients based on GSEA (left) and landscape analysis (right).

To evaluate the robustness of our gene signatures in discriminating between macrophage phenotypes, we analyzed the performance of our M(IFNγ + LPS, TNFα) and M(IL-4, IL-13) gene signatures in datasets from previously published studies that used controlled clinical conditions with the GSEA tool. Table 1 summarizes the description of the selected clinical studies where a macrophage polarization phenotype could be expected: *i*) the classically activated phenotype, using retrieved macrophages from bronchoalveolar lavage (BAL) in patients treated with lipopolysaccharide (LPS) (*GSE40885*^25^) and *ii*) the alternatively activated phenotype, using lung biopsies from asthmatic patients (*GSE41649*^26^). As expected, genes from the M(IFNγ + LPS, TNFα) set were significantly enriched in the LPS treated group (Fig. 3A). In addition, enrichment analysis of the asthmatic subjects was also significant in the M(L-4, IL-13) signature (Fig. 3B). Figure 3A,B (right) illustrate differential gene expression responses of the M(IFNγ + LPS, TNFα) and M(IL-4, IL-13) gene networks in both conditions. These results are consistent with the role played by LPS-induced IFNγ in pro-inflammatory macrophages or IL-4 driving an alternative activation state in the clinical context of asthma^12^,^27^,^28^.

We also tested 8 additional GEO datasets that used *in vitro* MDM or human cell lines under controlled culture environment, retrieving the expected results from polarization. As expected and in light of our findings, the M(IFNγ + LPS, TNFα) signature was enriched in genes altered under intracellular parasites and bacterial, or viral infection, as well as under pro-inflammatory conditions (LPS or IFNγ). By contrast, the M(IL-4, IL-13) gene signature was enriched in the context of IL-4, IL-10 or IL-13 stimulation (Supplementary Table S4).

Together, our results indicate that our macrophage signatures could characterize microarrays from *in vivo* specific pathological scenarios that take into account the complexity of the tissue components, as well as in *in vitro* factors that impact cultured primary human cells and human cell lines.

### Disease Outcomes Predictions and Functional Correlation in Complex Clinical Pathologies

As macrophages play a key role in determining the activation or resolution of immune responses and can determine the fate of a disease^29^, we evaluated the capacity of our macrophage phenotype signatures to anticipate the patient outcome or response to specific drug treatment (prognostic and predictive behavior of markers) based on the enrichment analysis of selected genes. We asked whether our M(IFNγ + LPS, TNFα) signature could be used to predict the complication of dengue infection into a hemorrhagic outcome, the drug response in HIV positive patients, and sepsis resolution in children.

**Figure 4:**
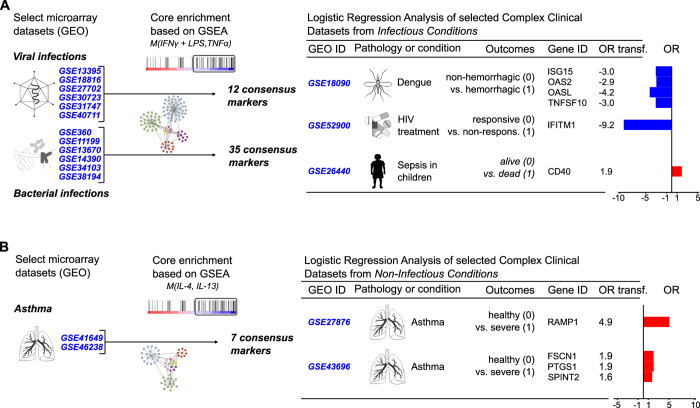
Prognostic and predictive values of selected components derived from M(IFNγ + LPS, TNFα) and M(IL-4, IL-13) signatures in complex clinical settings. Protocol design to select consensus responsive genes in infectious (**A**) and non-infections (**B**) conditions to interrogate different clinical datasets. The input lists for the consensus analysis comprised of 106 M(IFNγ + LPS, TNFα) and 58 M(IL-4, IL-13) genes. M(IFNγ + LPS, TNFα) list was interrogated for association with viral and bacterial (6 independent gene expression signatures each) infections, retrieving 12 and 35 consensus gene markers, respectively. M(IL-4, IL-13) list was interrogated for association with non-infectious conditions (2 independent gene expression signatures), retrieving 7 consensus gene markers (see Supplementary Table S5 & S6 for the complete description of datasets). Prognostic or predictive values of these markers were assessed by logistic regression analysis using selected clinical cohorts. Data were expressed as Odds Ratio (OR). (Drawings made by F. M. B-T).

To do so, we considered new microarray datasets in order to first identify a responsive set of genes presented in our macrophage signatures clustered by different pro-inflammatory scenarios (*e.g.*: viral and bacterial infections). We retrieved 6 datasets derived from viral and 6 from bacterial infections (Supplementary Table S5). For each analysis set, based on GSEA, we obtained a significant responsive gene list, considering an inclusion criterion for genes present in the core enrichment in more than 80% of cases. This procedure retrieved 12 genes in our M(IFNγ + LPS, TNFα) signature responsive to viral and 35 genes to bacterial infections (protocol design presented in Fig. 4A). With the virus and bacteria gene lists, we performed logistic regression analyses in order to associate gene expression with different disease outcomes. In this regard, we expressed our results as odds ratio (OR), which typically represents a measure of association between a predictor and an outcome. In our settings, a particular gene and a specific disease outcome prediction (*e.g*.: live versus death, disease severity or treatment response).

As depicted in Fig. 4A, when comparing controlled versus hemorrhagic dengue (*GSE18090*, unpublished data), we found that the individual expression of *ISG15*, *OASL*, *OAS2* and *TNFSF10* can be used to anticipate the complication status of dengue infection into a hemorrhagic outcome. Further combined analyses when all 4 genes were tested altogether was not able to improve OR. Similarly, the expression of *IFITM1* can discriminate the response of patients to HIV treatment (*GSE52900*^30^). From our bacterial infection list, we found that the expression of *CD40* was a predictor of death in septic children (*GSE26440*^31^).

We then searched for clinical conditions that could benefit from the same experimental approach using genes associated with the M(IL-4, IL-13) phenotype, such as asthma and pulmonary fibrosis (Supplementary Table S6), and constructed another list of genes with the GSEA tool (Fig. 4B). Data analysis enabled 2 comparisons and setup a threshold of 2/2 retrieved genes to be in the final list. In this new list, we obtained 7 genes. We performed logistic regression analyses and compared healthy versus severe asthmatic patients (*GSE27876*, unpublished data). We found that the expression of *RAMP1* was associated with the severity of asthma. In the same way (*GSE43696*^32^), the expression of three other genes *FSCN1*, *PSTG1* and *SPINT2* revealed an association with the development of severity of asthma (Data is summarized in Fig. 4B). Importantly, in all the 6 clinical datasets tested, we found a consistency in that the specified expressed genes were predicted by our created list M(IFNγ + LPS, TNFα) or M(L-4, IL-13).

## Discussion

Growing evidence defines macrophages as plastic cells with wide-ranging states of activation and concomitant expression of different markers, which are time and location dependent^2^,^33^. Because of pleiotropic actions of signaling through recognition receptors, secretion of cytokines, their essential role in activation of immunity or in resolution and the relation to disease outcome, published data has identified macrophages as key players in tissue homeostasis. Indeed, different from the initially proposed M1/M2 dichotomy^34^, plasticity and diversity have been extensively demonstrated as characteristic features of monocyte-macrophage differentiation^15^,^33^,^35^,^36^, pointing to the difficulty on defining complex profiles by a small and limited number of genes^33^. In this regard, conflicting and oversimplified data have been used to define different subsets. Thus, the generation of a simple and reliable framework to categorize major Mφ phenotypes in the context of complex clinical conditions would be extremely relevant to unravel different roles played by these cells in pathophysiological scenarios.

In the current study, we integrated transcriptome data using bioinformatics tools to generate gene expression profiling of macrophages, activated under precise and specific conditions, and created two macrophage molecular signatures from specific defined *in vitro* induced phenotypes, namely M(IFNγ + LPS, TNFα) and M(IL-4, IL-13). In line with these findings and the complexity associated with a wide range of evidence not easy to summarize, we validated our macrophage molecular signatures *in vitro* and in clinical samples from published microarray data. More importantly, we were also able to attribute prognostic and predictive values to some components of our signatures.

As expected, our study confirms that pro-inflammatory inductors such as IFNγ, LPS and TNFα are able to induce a macrophage phenotype distinct from that induced by cytokines as IL-4 and IL-13^14^,^15^,^37^. We confirmed the expression of well-established markers of classically (*e.g.,* *CD80, CXCL9, CXCL10, IL-15, ITGAL, TNFS10* and *STAT1*) and alternatively (*e.g.,* *ALOX15, CCL13, CCL17, F13A1, PTGS1*) activated macrophages markers that support the validity of our gene lists. In addition, our strategy retrieved several genes not previously used as M(IFNγ + LPS, TNFα) or M(IL-4, IL-13) markers that could be explored in the macrophage biology field.

In this regard, unexplored gene markers such as *LAG-3, LAMP3, OPTN* (optineurin) and *PIN-1* (peptidylprolyl cis/trans isomerase, NIMA-interacting 1) were highlighted in the M(IFNγ + LPS, TNFα) molecular signature. For example, LAG-3 is a high affinity ligand for MHC class II originally identified as a T and B-lymphocyte cell marker^38^,^39^. So far, no data has been shown describing its expression in circulating monocytes or activated macrophages. Activated LAG-3^ +^ lymphocytes present at sites of inflammation may reduce the differentiation of monocytes into macrophages or fully competent antigen-presenting dendritic cells, thus limiting the magnitude of the ongoing T-cell immune responses^40^. However, analysis of gene expression from three independent datasets (*GSE21548, GSE28785, GSE29628*) derived from pure monocytes cell lines (RAW264.7 and THP-1) from two different species (mouse and humans) showed consistently expression of LAG-3. At this point we cannot exclude the possibility that LAG-3 expression could be from contaminating cells in all three datasets, such as T and B lymphocytes, rather than from macrophages. Another newly classified gene was *LAMP3*, a well-established marker of mature dendritic cells^41^. Also, *OPTN* has a known role in vesicle trafficking and bacterial handling. However, their expression and role in human M(IFNγ + LPS, TNFα) macrophages remains unexplored. Interestingly, a recent work described the association of pro-inflammatory cytokine release deficiency in macrophages with reduced *OPTN* expression in a subset of patients with Crohn’s disease^42^. Finally, we were not able to find any study addressing the association between *PIN-1* and macrophages. As these examples, several other genes retrieved in the M(IFNγ + LPS, TNFα) list have not yet been explored as potential macrophage markers. Thereby, our list suggest a great variety of genes to be studied and used as new M(IFNγ + LPS, TNFα) macrophage markers.

In addition, the M(IL-4, IL-13) molecular signature retrieved genes such as *FZD2*, *CLIC2*, *EMILIN2* (elastin microfibril interfacer 2), *CDR2L* (cerebellar degeneration-related protein 2-like), *CMTM8* (CKLF-like MARVEL transmembrane domain containing 8) that has not been fully explored as potential human macrophage markers. In this sense, *FZD2* has a potential role in macrophage-regulated angiogenesis, as proposed by Newman *et al.*^43^. *CLIC2* is a chloride intercellular channel that is not reported as M(IL-4, IL-13) marker, but few evidences suggest that nuclear translocation of *CLIC4* regulates macrophage deactivation^44^. Another poorly explored gene in human macrophages, *EMILIN2* gene was not explored, but one study with mouse macrophages established a correlation between EMILIN2 protein and thrombosis^45^. Finally to our knowledge, there are no association between *CDR2L* and *CMTM8* genes and macrophages.

Because we were interested in exploring the potential application of our framework in profiling macrophages’ phenotypes, we generated logistic regression models to associate the consensus markers with diseases outcomes. In this regard, we show that a set of selected markers is able to predict patients’ outcomes when dissimilar pathologies were grouped according to infectious (*e.g.*: dengue, HIV, and sepsis) or non-infectious conditions (*e.g.*: asthma) (Fig. 4).

Our initial analysis of viral infection responsive genes derived from our M(IFNγ + LPS, TNFα) signature retrieved *TNFSF10*, *ISG15*, *OAS2* and *OASL*. For example, we found that the expression levels of *TNFSF10* (tumor necrosis factor (ligand) superfamily, member 10, commonly known as TRAIL), obtained from the M(IFNγ + LPS, TNFα) signature, could discriminate the severe cases of hemorrhagic dengue. The protective role of *TNFSF10*, as apoptosis inducer, in dengue severity or symptoms’ complications has already been identified^46^,^47^. Serum from patients with dengue had significantly increased TNFSF10 protein levels^48^. Its antiviral action has been demonstrated in various cells, including in *ex vivo* infected monocytes and macrophages^49^. Another gene that was shown to protect from dengue severity was Interferon-Stimulated Gene 15 (*ISG15*)^50^,^51^. Regardless, our logistic regressions analyses demonstrates that the expression of *ISG15* can predict the course of dengue patients into a more severe outcome. Indeed, a recent published study^52^ with infected cultured cell lines implicated *ISG15* in dengue virus 2 replication inhibition. Moreover, two members of the OAS (2,5-oligoadenylate synthetase) gene family, *OAS2* and *OASL*, also contribute to the antiviral response. Two recent studies have demonstrated an association between *OAS2* haplotypes and differential susceptibility to clinical outcomes of dengue virus infection^53^,^54^. In addition, other members of the OAS gene family, as *OAS1* p42, *OAS1* p46, and *OAS3* p100 have been shown to have antiviral effects in dengue complication^55^. Therefore, these IFN-induced proteins may play important roles in the antiviral response and can be addressed as promising targets poorly explored in the literature for the management of dengue infection.

Other applications of our molecular signature could be to anticipate clinical response to treatment. In this aspect, we found that *IFITM1* (interferon-induced transmembrane 1) is associated with better response to HIV treatment, presenting an OR of 9.4. *IFITM1* has antiviral action already demonstrated in highly pathogenic human virus^56^ and has been associated with cell-to-cell HIV-1 transmission^57^, but how *IFITM1* contributes to treatment resistance is still unclear. We went further and analyzed the bacterial infection responsive genes derived from our M(IFNγ + LPS, TNFα) signature and applied in predicting sepsis outcome. We found that the expression of *CD40*, a molecular target that had already been linked with inflammation^58^, have prognostic role for septic patients. Altogether these results emphasize the robustness of our genes signatures and demonstrate that our analysis framework was able to predict and validate the presence of already known genes or find new candidates to further explore association between expression, biological function and clinical outcome.

In the same context, we applied our M(IL-4, IL-13) signature to predict outcomes in asthma cohorts. Asthma is a heterogeneous disease that is classified phenotypically as mild, moderate, or severe^59^. We found that *RAMP1*, *FSCN1*, *PSTG1* and *SPINT2* genes could be associated with the development of severity of asthma. However, no studies showed an association of *FSCN1* (fascin actin-bundling protein 1) and *SPINT2* (serine peptidase inhibitor, Kunitz type, 2) with the evolution of asthma severity.

In conclusion, we proposed a consensus collection of markers describing major macrophages’ activation phenotypes, namely M(IFNγ + LPS, TNFα) and M(IL-4, IL-13), able to characterize robustly controlled *in vitro* and *in vivo* scenarios for macrophage induction. Since the description of macrophage activation is currently contentious and confusing^15^,^33^, our study provides a framework to guide the interrogation of macrophage phenotypes in the context of health and disease. Despite further studies being necessary to understand the role played by retrieved genes, the approach described unraveled new gene candidate markers for diverse clinical settings such as dengue infections, asthma and sepsis resolution.

## Methods

The methods were carried out in accordance with the approved guidelines.

### Ethics

This study was approved by the Ethics Committee of PUCRS (No. 06/03537). All participants provided written informed consent for blood collection and research.

### Microarray Datasets and Macrophage Phenotypes’ Signatures

Microarray expression profiles were extracted from public available Gene Expression Omnibus (GEO) NCBI database (http://www.ncbi.nlm.nih.gov/geo/). The selected GSE datasets are presented in Supplementary Table S1. Search criteria included Medical Subject Heading (MeSH) terms for “monocytes-derived macrophages” and “*Homo sapiens*” and “macrophages polarization”.

GEO2R tool (www.ncbi.nlm.nih.gov/geo/geo2r) was used to identify differential gene expression to obtain classically activated M(IFNγ + LPS,TNFα) and alternatively activated M(IL-4, IL-13) phenotypes signatures^60^. We extracted two lists of genes with a significant different expression in the two polarized Mφ discrete phenotypes with adjusted *P-*value ≤ 0.0001 Student’s *t*-test and Benjamini & Hochberg false-discovery rate (FDR) correction. Finally, an integrated gene list for each phenotype was created where all the pre-selected differentially expressed genes that were consistently present in the three datasets were included (protocol design in Fig. 1A).

### Gene Set Enrichment Analyses (GSEA) and Network Construction

We explored the robustness of our gene signatures using GSEA method (http://www.broadinstitute.org/gsea/index.jsp)^61^. In this regard, genes are ranked based on the correlation between their expression and their class distinction, M1-*like* or M2-*like*, by using any suitable metric. In that case, the method evaluates if an *a priori* defined set of genes (*e.g.:* M(IFNγ + LPS, TNFα) and M(IL-4, IL-13) gene signatures) is randomly distributed or is primarily associated with a tested class^61^. Expression networks of differential signatures graphs and illustrative landscape maps were constructed and edited using free academic Medusa^®^ (https://sites.google.com/site/medusa3visualization)^62^, and ViaComplex^®^ (http://lief.if.ufrgs.br/pub/biosoftwares/viacomplex)^24^ softwares.

### Logistic Regression

Logistic regression models associate binary responses with continuous variables. Specifically, we applied a bias-reduced logistic regression to test gene expressions against defined pathological outcomes in an attempt to identify promising markers from our macrophage signatures. Firth’s penalized-likelihood logistic regression was originally developed to reduce the bias of maximum likelihood estimates and provide a good solution to monotone likelihoods (http://www.meduniwien.ac.at/user/georg.heinze/techreps/tr2_2004.pdf) (http://www.jstor.org/stable/2336755?origin=JSTOR-pdf&seq=1#page_scan_tab_contents). The regressions were modeled using R statistical environment (http://www.R-project.org).

### Cell Cultures and Macrophage Differentiation

PBMC from healthy individuals (n = 5) were isolated by Histopaque^®^ gradient (d = 1.077) (Sigma Aldrich, MO, USA) according to manufacturer’s instructions. Monocytes were purified from freshly isolated PBMC using monoclonal CD14 antibody-conjugated microbeads (Miltenyc Biotec, Germany). Purity was >98%. Monocytes were cultured in RPMI-1640 media (Invitrogen, CA, USA), supplemented with 10% heat-inactivated fetal bovine serum (FBS) (Invitrogen), 2 mM L-glutamine (Invitrogen), 100 U/mL penicillin and 100 mg/mL streptomycin (Invitrogen) (RPMI 10% FBS) at 37 °C in 5% CO_2_ humidified air. For *in vitro* differentiation of MDM, monocytes were incubated with RPMI (10% FBS) supplemented with Macrophage Colony Stimulating Factor (M-CSF) (50 ng/mL) (Peprotech, USA) for 7 days. For differential polarization, Mφ were supplemented with IFNγ (20 ng/mL) (Peprotech, USA) and LPS (100 ng/mL) (Sigma-Aldrich) or IL-4 (20 ng/mL) (Peprotech, USA) for additional 18 h, respectively.

The THP-1 (human acute monocytic leukemia) and U-937 (human histiocytic lymphoma) cells lines were obtained from Rio de Janeiro Cell Bank (www.bcrj.org.br) and maintained in RPMI-1640 media as mentioned above. Cell lines were differentiated using phorbol 12-myristate 13-acetate (PMA) (Sigma Aldrich), 20 nM and 10 nM for THP-1 or U-937 cells, for 72 h. PMA treated cells were polarized for additional 24 h by incubation with IFNγ (20 ng/mL) + LPS (100 ng/mL) or IL-4 (20 ng/mL) or IL-13 (20 ng/mL) for M(IFNγ + LPS), M(IL-4) or M(IL-13) phenotypes.

### RNA Isolation and RT-qPCR

Gene expression analysis was performed using gene-specific primers designed with IDT Design Software (Integrated DNA Technologies Inc., CA, USA) (Supplementary Table S7). Total RNA (1.2 μg) was isolated from M(IFNγ + LPS) and M(IL-4) cells using Trizol Reagent (Invitrogen). Reverse transcription was performed with M-MLV Reverse Transcriptase (Sigma-Aldrich) and random nonamers (Sigma-Aldrich) primers. Real-time PCR reactions were carried out in Step One Plus real-time cycler (Applied-Biosystem, NY, USA) using Taq Polimerase (Sigma-Aldrich) and SYBR green. Gene expression was quantified by the comparative cycle threshold method (ΔΔCT) and normalized using the housekeeping gene TATA binding protein (TBP). Melting curves were used to monitor unspecific amplification products.

## Additional Information

**How to cite this article**: Becker, M. *et al.* Integrated Transcriptomics Establish Macrophage Polarization Signatures and have Potential Applications for Clinical Health and Disease. *Sci. Rep.* **5**, 13351; doi: 10.1038/srep13351 (2015).

## Supplementary Material

Supplementary Information

## Figures and Tables

**Table 1:** Gene Set Enrichment Analysis (GSEA) of M(IFNγ + LPS, TNFα) and M(IL-4, IL-13) gene networks in clinical samples.

**GEO ID**	**Cohort Description**	**Experimental Groups**	**Reference**
*GSE40885*	Sterile saline was instilled into a lung segment by bronchoscope, followed of instillation of LPS into the contralateral lung for 6 hours. After, a bilateral bronchoalveolar lavage was performed and transcriptional profiling was done on alveolar Mφ.	Alveolar Mφ from saline (n = 7) vs. LPS-treated (n = 7).	Reynier, F. *et al.* Gene expression profiles in alveolar macrophages induced by lipopolysaccharide in humans. Molecular Medicine 2012 (18):1303–1311.
*GSE41649*	Transcriptional profiling of bronchial biopsy of healthy and asthmatic subjects.	Healthy (n = 4) vs. asthmatic patients (n = 4).	Chamberland, A. *et al.* A comparison of two sets of microarray experiments to define allergic asthma expression pattern. Experimental lung research 2009 (35): 399–410.

Abbreviations: IFNγ, interferon-gamma; IL-4, interleukin-4; IL-13, interleukin-13; LPS, lipopolysaccharide; Mφ, macrophages; TNFα, tumor necrosis factor-alpha.
