# Supplementary material for: Integrated Transcriptomics Establish Macrophage Polarization Signatures and have Potential Applications for Clinical Health and Disease
Source: Sci Rep. 2015 Aug 25;5:13351. doi: 10.1038/srep13351 (PMC4548187; doi:10.1038/srep13351)
Supplement: Supplementary Information [file srep13351-s1.pdf]

Supplementary Information

Research Article

Integrated Transcriptomics Establish Macrophage Polarization Signatures and have Potential Applications for Clinical Health and Disease

Authors

Matheus Becker, Marco A. De Bastiani, Mariana M. Parisi, Fátima T. C. R. Guma, Melissa M. Markoski, Mauro A. A. Castro, Mark H. Kaplan, Florencia M. Barbé-Tuana & Fábio Klamt.

Supplementary Table S1: Summary description of datasets used to create the M(IFNγ + LPS, TNFα) and M(IL-4, IL-13) signatures.

GEO ID	Summary Description
GSE5099	Peripheral monocytes were induced to mature macrophages with M-CSF. Cells were then activated with IFNγ and LPS or IL-4.
GSE35449	Analysis of human M0, M1 (IFNγ, LPS and TNFα stimulus), and M2 (IL-4 and IL-13 stimulus)-like macrophages derived from peripheral blood.
GSE36537	MDM were stimulated with IFNγ to M1-like phenotype and IL-4 for M2-like phenotype.

Abbreviations: IFNγ, interferon-gamma; IL-4, interleukin-4; IL-10, interleukin-10; IL-13, interleukin-13; LPS, lipopolysaccharide; M0, unstimulated macrophage; M-CSF, macrophage colony stimulating factor; MDM, monocyte derived macrophages.

Supplementary Table S2: M(IFNγ + LPS, TNFα) gene list.

GENE	Description	Gene Classification*
ADAM28	ADAM metallopeptidase domain 28	Matrix
AIM2	absent in melanoma 2	Others
ANKRD22	ankyrin repeat domain 22	Others
APOBEC3A	apolipoprotein B mRNA editing enzyme, catalytic polypeptide-like 3A	Enzyme
APOL1	apolipoprotein L, 1	Others
APOL3	apolipoprotein L, 3	Others
BATF2	basic leucine zipper transcription factor, ATF-like 2	Transcription factor
C1R	complement component 1, r subcomponent	Enzyme
C1S	complement component 1, s subcomponent	Enzyme
CCL19	chemokine (C-C motif) ligand 19	Chemokine
CD38	CD38 molecule	Receptor
CD40	CD40 molecule, TNF receptor superfamily member 5	Receptor
CD80	CD80 molecule	Receptor
CFB	complement factor B	Others
CLEC4D	C-type lectin domain family 4, member D	Receptor
CXCL10	chemokine (C-X-C motif) ligand 10	Chemokine
CXCL9	chemokine (C-X-C motif) ligand 9	Chemokine
CYBB	cytochrome b-245, beta polypeptide	Others
DUSP10	dual specificity phosphatase 10	Enzyme
DUSP6	dual specificity phosphatase 6	Enzyme
ETV7	ets variant 7	Transcription factor
FAM49A	family with sequence similarity 49, member A	Others
FAM65B	family with sequence similarity 65, member B	Others
FCGR1B	Fc fragment of IgG, high affinity Ib, receptor (CD64)	Recptor
FPR2	formyl peptide receptor 2	Receptor
GADD45G	growth arrest and DNA-damage-inducible, gamma	Others
GBP1	guanylate binding protein 1, interferon-inducible, 67kDa	Others
GBP2	guanylate binding protein 2, interferon-inducible	Others
GBP4	guanylate binding protein 4	Others
GBP5	guanylate binding protein 5	Others
GCH1	GTP cyclohydrolase 1	Enzyme
GK	glycerol kinase 3 pseudogene; glycerol kinase	Enzyme
GPR84	G protein-coupled receptor 84	Receptor

GUCY1A3	guanylate cyclase 1, soluble, alpha 3	Enzyme
HERC5	hect domain and RLD 5	Enzyme
HESX1	HESX homeobox 1	Transcription factor
HLA-F	major histocompatibility complex, class I, F	Receptor
IFI27	interferon, alpha-inducible protein 27	Others
IFI35	interferon-induced protein 35	Others
IFI44L	interferon-induced protein 44-like	Others
IFIH1	interferon induced with helicase C domain 1	Receptor
IFIT2	interferon-induced protein with tetratricopeptide repeats 2	Others
IFIT3	interferon-induced protein with tetratricopeptide repeats 3	Others
IFITM1	interferon induced transmembrane protein 1 (9-27)	Others
IFITM2	interferon induced transmembrane protein 2 (1-8D)	Others
IL15	interleukin 15	Cytokine
IL15RA	interleukin 15 receptor, alpha	Receptor
IL32	interleukin 32	Cytokine
INHBA	inhibin, beta A	Others
IRF1	interferon regulatory factor 1	Transcription factor
IRF7	interferon regulatory factor 7	Transcription factor
ISG15	ISG15 ubiquitin-like modifier	Others
ISG20	interferon stimulated exonuclease gene 20kDa	Enzyme
ITGAL	integrin, alpha L (antigen CD11A (p180), lymphocyte function-associated antigen 1; alpha polypeptide)	Receptor
ITGB7	integrin, beta 7	Receptor
LAG3	lymphocyte-activation gene 3	Others
LAMP3	lysosomal-associated membrane protein 3	Others
LIMK2	LIM domain kinase 2	Enzyme
LRRK2	leucine-rich repeat kinase 2	Enzyme
MUC1	mucin 1, cell surface associated	Others
MX1	myxovirus (influenza virus) resistance 1, interferon-inducible protein p78 (mouse)	Others
NAMPT	nicotinamide phosphoribosyltransferase	Enzyme
NFKBIZ	nuclear factor of kappa light polypeptide gene enhancer in B-cells inhibitor, zeta	Transcription factor ??
OAS1	2',5'-oligoadenylate synthetase 1, 40/46kDa	Enzyme
OAS2	2'-5'-oligoadenylate synthetase 2, 69/71kDa	Enzyme
OAS3	2'-5'-oligoadenylate synthetase 3, 100kDa	Enzyme
OASL	2'-5'-oligoadenylate synthetase-like	Enzyme
OPTN	optineurin	Transcription factor
PAG1	phosphoprotein associated with glycosphingolipid microdomains 1	Others
PARP14	poly (ADP-ribose) polymerase family, member 14	Enzyme
PCNX	pecanex homolog (Drosophila)	Others

PDE4B	phosphodiesterase 4B, cAMP-specific (phosphodiesterase E4 dunce homolog, Drosophila)	Enzyme
PIM1	pim-1 oncogene	Enzyme
PRKAR2B	protein kinase, cAMP-dependent, regulatory, type II, beta	Others
PSMB9	proteasome (prosome, macropain) subunit, beta type, 9 (large multifunctional peptidase 2)	Enzyme
PTGS2	prostaglandin-endoperoxide synthase 2 (prostaglandin G/H synthase and cyclooxygenase)	Enzyme
RARRES3	retinoic acid receptor responder (tazarotene induced) 3	Others
RCN1	reticulocalbin 1, EF-hand calcium binding domain	Others
RHBDF2	rhomboid 5 homolog 2 (Drosophila)	Others
RSAD2	radical S-adenosyl methionine domain containing 2	Others
SAT1	spermidine/spermine N1-acetyltransferase 1	Enzyme
SCO2	SCO cytochrome oxidase deficient homolog 2 (yeast)	Others
SEPT4	septin 4	Others
SERPING1	serpin peptidase inhibitor, clade G (C1 inhibitor), member 1	Others
SLAMF7	SLAM family member 7	Receptor
SLC22A15	solute carrier family 22, member 15	Others
SLC25A28	solute carrier family 25, member 28	Others
SLC31A2	solute carrier family 31 (copper transporters), member 2	Others
SLC6A12	solute carrier family 6 (neurotransmitter transporter, betaine/GABA), member 12	Others
SLC7A5	solute carrier family 7 (cationic amino acid transporter, y+ system), member 5	Others
SNTB1	syntrophin, beta 1 (dystrophin-associated protein A1, 59kDa, basic component 1)	Others
SNX10	sorting nexin 10	Others
SOCS3	suppressor of cytokine signaling 3	Trancription Factor
SOD2	superoxide dismutase 2, mitochondrial	Enzyme
STAT1	signal transducer and activator of transcription 1, 91kDa	Trancription Factor
STAT3	signal transducer and activator of transcription 3 (acute-phase response factor)	Trancription Factor
STX11	syntaxin 11	Others
TAP1	transporter 1, ATP-binding cassette, sub-family B (MDR/TAP)	Others
TNFAIP6	tumor necrosis factor, alpha-induced protein 6	Others
TNFSF10	tumor necrosis factor (ligand) superfamily, member 10	Cytokine
TRIM69	tripartite motif-containing 69	Enzyme
UBE2L6	ubiquitin-conjugating enzyme E2L 6	Enzyme
USP18	ubiquitin specific peptidase 18	Enzyme
VAMP5	vesicle-associated membrane protein 5 (myobrevin)	Others
WARS	tryptophanyl-tRNA synthetase	Enzyme
XRN1	5'-3' exoribonuclease 1	Enzyme

* Gene classification according to Murray et al.[15].

Supplementary Table S3: M(IL-4, IL-13) gene list.

GENE	Description	Gene classification*
ADAM19	ADAM metallopeptidase domain 19 (meltrin beta);	Matrix
ALOX15	arachidonate 15-lipoxygenase;	Enzyme
ARRB1	arrestin, beta 1;	Transcription factor
BZW2	basic leucine zipper and W2 domains 2;	Others
CARD9	caspase recruitment domain family, member 9;	Others
CCL13	chemokine (C-C motif) ligand 13;	Chemokine
CCL17	chemokine (C-C motif) ligand 17;	Chemokine
CCL23	chemokine (C-C motif) ligand 23;	Chemokine
CD1A	CD1a molecule;	Receptor
CD1C	CD1c molecule;	Receptor
CD1E	CD1e molecule; T-cell surface glycoprotein CD1e;	Receptor
CDR2L	cerebellar degeneration-related protein 2-like;	Others
CHN2	chimerin (chimaerin) 2; GTPase-activating protein for p21-rac;	Others
CLEC4A	C-type lectin domain family 4, member A;	Receptor
CLIC2	Chloride intracellular channel 2	Others
CMTM8	CKLF-like MARVEL transmembrane domain containing 8;	Cytokine
CRIP1	cysteine-rich protein 1 (intestinal);	Others
CTSC	cathepsin C; Thiol protease. endopeptidase.	Enzyme
DUSP22	Dual specificity phosphatase 22;	Enzyme
EMILIN2	elastin microfibril interfacer 2;	Matrix
ESPNL	espin-like;	Others
F13A1	coagulation factor XIII, A1 polypeptide;	Matrix
FOXQ1	forkhead box Q1;	Transcription Factor
FSCN1	fascin homolog 1, actin-bundling protein (Strongylocentrotus purpuratus);	Others
FZD2	frizzled homolog 2 (Drosophila); Receptor for Wnt proteins;	Receptor
GALNTL4	UDP-N-acetyl-alpha-D-galactosamine:polypeptide N-acetylgalactosaminyltransferase-like 4;	Enzyme
GATM	glycine amidinotransferase (L-arginine:glycine amidinotransferase);	Enzyme
GPD1L	glycerol-3-phosphate dehydrogenase 1-like;	Enzyme
GSTP1	glutathione S-transferase pi 1;	Enzyme
ITM2C	integral membrane protein 2C;	Others
KCNK6	potassium channel, subfamily K, member 6;	Others
MAOA	monoamine oxidase A;	Enzyme
MAP4K1	mitogen-activated protein kinase kinase kinase kinase 1;	Enzyme
MAPKAPK3	mitogen-activated protein kinase-activated protein kinase 3;	Enzyme
MFNG	MFNG O-fucosylpeptide 3-beta-N-acetylglucosaminyltransferase;	Enzyme

MS4A6A	membrane-spanning 4-domains, subfamily A, member 6A;	Receptor
NMNAT3	nicotinamide nucleotide adenylyltransferase 3; n	Enzyme
OSBPL7	oxysterol binding protein-like 7;	Others
P2RY11	purinergic receptor P2Y, G-protein coupled, 11;	Receptor
PALLD	palladin, cytoskeletal associated protein; a variety of cell types;	Others
PAQR4	progestin and adipoQ receptor family member IV;	Receptor
PELP1	proline, glutamate and leucine rich protein 1;	Transcription factor
PLAU	plasminogen activator, urokinase;	Enzime
PON2	paraoxonase 2;	Enzime
PPP1R14A	protein phosphatase 1, regulatory (inhibitor) subunit 14A;	Others
PTGS1	prostaglandin-endoperoxide synthase 1;	Enzyme
RAMP1	receptor (G protein-coupled) activity modifying protein 1;	Receptor
REPS2	RALBP1 associated Eps domain containing 2;	Others
RGS18	regulator of G-protein signaling 18;	Others
RRS1	RRS1 ribosome biogenesis regulator homolog (S. cerevisiae);	Others
S100A4	S100 calcium binding protein A4;	Others
SEC14L5	SEC14-like 5 (S. cerevisiae);	Others
SHPK	Sedoheptulokinase;	Enzyme
SPINT2	serine peptidase inhibitor, Kunitz type, 2;	Others
TGFBI	transforming growth factor, beta-induced, 68kDa;	Matrix
TMEM97	transmembrane protein 97;	Others
VCL	vinculin; Involved in cell adhesion;	Matrix
ZNF789	zinc finger protein 789;	Transcription factor

* Gene classification according to Murray et al.[15].

Supplementary Table S4: Features and results obtained of GSE datasets that used classical activators and different conditions.

GEO ID	COMPARISON	SAMPLE SIZE	RESULT	P
GSE360	Monocyte-derived macrophages (MΦ) were exposed to five different pathogens - *Mycobacterium tuberculosis* and to phylogenetically distinct protozoan *Leishmania major, L. donovani, Toxoplasma gondii* and helminth *Brugia malayi.*	MΦ unstimulated (2 samples) x MΦ stimulated with five different pathogens (2 samples)	M(IFNγ + LPS, TNFα) network genes were enriched in macrophages exposed to pathogens.	$P < 0.001$
GSE1432	Unstimulated microglia cells x microglia cells stimulated with IFN-gamma for 1h and 24h	4 samples for control, 1h and 24h	M(IFNγ+LPS, TNFα) genes were enriched in microglia cells stimulated with IFN-gamma in both times	$P < 0.001$ for both times
GSE15038	PMA stimulated only x PMA and LPS stimulated U937 cells	1 for both samples	M(IFNγ + LPS, TNFα) genes were enriched in LPS stimulated cells	$P = 0.001$
GSE25289	Unstimulated microglia cells x stimulated with Poly I:C	5 samples for both	M(IFNγ + LPS, TNFα) genes were enriched in Poly I:C	$P < 0.001$
GSE29628	Control x THP-1 cells infected with different *W-Beijing Mycobacterium tuberculosis* strains of different sublineages	1 for control and 13 for *M. tuberculosis*	M(IFNγ + LPS, TNFα) genes were enriched in *M. tuberculosis* stimulated cells	$P < 0.001$
GSE32164	Unstimulated MDMs (M0) x Stimulated MDMs with IL-4	3 for both samples	M(IL4, IL-13) genes were enriched in IL-4 stimulated macrophages	$P < 0.001$
GSE49240	MDMs stimulated cells; IL-4 x IFN-gamma; MDMs stimulated cells; IL-10 x IFN-gamma	2 for IL-4, Il-10 and IFN-gamma	M(IFNγ + LPS, TNFα) genes were enriched in IFN-gamma stimulated cells in both comparisons. M(IL-4, IL-13) genes were enriched in IL-4 and IL-10 stimulated cells	$P < 0.001$ for all comparisons
GSE51446	MDMs stimulated cells; IL-4 x LPS; MDMs stimulated cells; IL-10 x LPS; MDMs stimulated cells; IL-13 x LPS	1 for IL-4, IL-10, IL-13 and LPS	M(IFN-γ + LPS, TNF-α) genes were enriched in LPS stimulated cells. M(IL-4,IL13) genes were enriched in IL-4, IL-10 and IL-13 stimulated cells	$P < 0.001$ for LPS x IL-4<br>$P < 0.05$ for LPS x IL-10 and IL-13.

Abbreviations: IFNγ, interferon-gamma; IL-4, interleukin-4; IL-10, interleukin-10; IL-13, interleukin-13; LPS, lipopolysaccharide; M. tuberculosis, Mφ, macrophages; MDM, monocyte-derived macrophage; *Mycobacterium tuberculosis*; PMA, phorbol myristate acetate; T. gonddi, *Toxoplasma gondii*.

Supplementary Table S5: Description of datasets used to create the virus and bacterial lists.

GEO ID	Description
	Bacterial Datasets
GSE360	MDM generated *in vitro* from the same individual blood donors were exposed to five different pathogens, and gene expression profiles were assessed by microarray analysis. Responses to *Mycobacterium tuberculosis* and to phylogenetically distinct protozoan (*Leishmania major*, *L. donovani, Toxoplasma gondii*) and helminth (*Brugia malayi*) parasites were examined, each of which produces chronic infections in humans yet vary considerably in the nature of the immune responses they trigger.
GSE11199	Peripheral blood leucocytes were separated from buffy coats of three healthy blood donors and cells were differentiated for 14 days before use. Differentiated macrophages were infected with H1N1 and H5N1 at MOI 2. Total RNA was extracted from cells after 1, 3, and 6h post-infection, and gene expression profiling was performed using an Affymetrix Human Gene 1.0 ST microarray platform.
GSE13670	Human MDM were separated from fractions of PBMC obtained from the blood of healthy donors. Control and *S. aureus*-exposed macrophages were incubated at 37 °C for 8, 24, or 48 hours.
GSE14390	HAM was exposed to *Bacillus anthracis* Sterne spores at a MOI of 1 for 6 hours. RNA was extracted from HAM and analyzed by the Affymetrix Human Genome U133 Plus 2.0 Array. The transcriptional profile of *B. anthracis* spore-treated HAM was compared with mock infected cells, and differentially expressed genes were identified.
GSE34103	Human macrophages were generated from PBMC obtained by Pancoll density centrifugation from buffy coats of healthy donors. Subsequently, macrophages were infected with *L. monocytogenes* using a MOI of 10. Total RNA isolated from *L. monocytogenes* infected macrophages 24 hours post-infection was compared to control non infected macrophages
GSE38194	MDM (4×10^5 cells per assay) were incubated with *Orientia tsutsugamushi* at a bacterium-to-cell ratio of 20:1 for 8 hours. RNA samples (four samples per experimental condition) were processed for microarray analysis.
	Virus Datasets
GSE13395	An HIV-1 spreading infection was established in primary human macrophages. RNA was extracted from both viral- and mock-infected macrophages cultures over 7 days and hybridized to Affymetrix HG-U95Av2 GeneChips for analysis.
GSE18816	Peripheral-blood leucocytes were separated from buffy coats of three healthy blood donors and cells were differentiated for 14 days before use. Differentiated macrophages were infected with H1N1 and H5N1 at a MOI of 2. Total RNA was extracted from cells after 1, 3, and 6h post-infection, and gene expression profiling was performed using an Affymetrix Human Gene 1.0 ST microarray platform.
GSE27702	Human monocytes were isolated from buffy coats of unrelated, healthy German blood donors and human macrophages were obtained by cultivating isolated monocytes in Teflon bags in RPMI-1640 medium, supplemented with 1% glutamine, 1% penicillin-streptomycin, and 10% human AB serum provided by the Department of Transfusion Medicine, University of Muenster (Germany). Medium was substituted every 3 days, and cells were used in experiments on Day 7. Macrophages were infected with low (PR/8) and high pathogenic influenza viruses (FPV and H5N1).
GSE30723	Primary cultured cells were infected with or without influenza virus PR/8 at MOI of 0.5. At 4h and 24h, total RNA were isolated for microarray experiments.
GSE31747	Primary human macrophages from three donors (D1, D2 and D3) were harvested at 1 h and 6 h after *in vitro* exposure to purified Ebola virions and compared to RNA from mock-exposed cells derived from the same donors.

GSE40711	Human MDM were mock-infected, or infected with H5N1 strains IDN3006 (low cytokine inducer), VN3028IIcl2 (high cytokine inducer), or IDN3006/cl2PA at a MOI of 2. At 6 hours post-infection, cells were harvested and subjected to microarray analysis (three technical replicates per each group).

Abbreviations: HAM, human alveolar macrophages; Mφ, macrophages; MDM, monocyte-derived macrophage; MOI, multiplicity of infection; PBMC, Peripheral blood mononuclear cells; PMA, Phorbol myristate acetate.

Supplementary Table S6: Summary description of datasets used to create the M(IFNγ + LPS, TNFα) and M(IL-4, IL-13) signatures.

GEO ID	Summary Description
GSE41649	This dataset aimed to demonstrate the relevance and validity of microarrays in the definition of allergic asthma expression pattern. The authors compared the transcript expressions of bronchial biopsy of 2 different microarray experiments done 2 years apart, both including non-allergic healthy and allergic asthmatic subjects (n = 4 in each experiment). The results demonstrated the relevance of microarray experiments using bronchial tissues in allergic asthma.
GSE46238	Sputum cells collected before (visit 2) and after (visit 4) allergen challenge in asthma patients were isolated and RNA purified for analysis on gene expression arrays.

Abbreviations: IFNγ, interferon-gamma; IL-4, interleukin-4; IL-13, interleukin-13; LPS, lipopolysaccharide.

Supplementary Table S7: Primers used for M(IFNγ + LPS) and M(IL-4) analyses in MDM and cell lines.

Gene	Access	Primers Sequences		Amplicon
		Forward	Reverse	
CXCL9	NM_002416.1	GGACTATCCACCTACAATCCTTG	TTTTAATCAGTTCCTTCACATCTGC	147 bp
CXCL10	NM_001565.3	ACTCTAAGTGGCATTCAAGGAG	ACGTGGACAAAATTGGCTTG	135 bp
IL1-beta	NM_000576.2	TGGCCCTAAACAGATGAAGTG	GAAGGTGCTCAGGTCATTCTC	199 bp
IL-15	NM_172175.2	CTGATCATCCTAGCAAACAACAG	GGACAATATGTACAAAACTCTGCAA	127 bp
STAT1	NM_007315.3	TGACTCAAAATTCCTGGAGCAG	AGGTCATGAAAACGGATGGTG	150 bp
TNF-alpha	NM_000594.3	TCTCTCTAATCAGCCCTCTGG	GCTTGAGGGTTTGCTACAAC	94 bp
ALOX15	NM_001140.3	GAGGTCAGGTTCCCTTGTTAC	CTTCTCTCTTCCAGCTCTTCTTC	137 bp
CCL13	NM_005408.2	CAGAGGCTGAAGAGCTATGTG	CGGCCCAGGTGTTTCATATAA	137 bp
CCL17	NM_002987.2	CCCCTTAGAAAGCTGAAGACG	TCAAGGCTTTGCAGGTATTTAAC	149 bp
F13A	NM_000129.3	TCCTGGAGTAACAAGACCAATG	CATACACATGTCTCAGGGAGTC	149 bp
TGF-beta	NM_000660.5	TGCTGTCTCCATGTTTGAT	TCTCTGCTCCCCACCTCTA	86 bp

Abbreviations: BP, base pairs; MDM, monocyte-derived macrophage.
